# The Uremic Toxin Homocysteine Exacerbates the Brain Inflammation Induced by Renal Ischemia-Reperfusion in Mice

**DOI:** 10.3390/biomedicines10123048

**Published:** 2022-11-25

**Authors:** Eun Jung Park, Jihyun Je, Theodomir Dusabimana, Seung Pil Yun, Hye Jung Kim, Hwajin Kim, Sang Won Park

**Affiliations:** 1 Department of Pharmacology, Institute of Health Sciences, College of Medicine, Gyeongsang National University, Jinju 52727, Republic of Korea; 2 Department of Convergence Medical Sciences, Graduate School, Gyeongsang National University, Jinju 52727, Republic of Korea

**Keywords:** acute kidney injury, astrocytes, brain, homocysteine, inflammation

## Abstract

Homocysteine (Hcy), a homologue of cysteine, is biosynthesized during methionine metabolism. Elevated plasma Hcy is associated with glomerular injury and considered as a risk factor for renal dysfunction, predicting incident chronic kidney disease. Hcy promotes oxidative stress, inflammation, and endothelial dysfunction. Acute kidney injury (AKI) is defined as a sudden decline in renal function and is important clinically due to the high mortality rate in AKI patients with multiple organs failure, including the brain. However, the cytotoxic role of Hcy on the brain following AKI is not directly shown. In this study, C57BL/6 mice were subjected to renal ischemia reperfusion (IR), one of the causes of AKI, and treated with vehicle or Hcy (0.2 mg/kg) to analyse the brain inflammation. IR mice showed a significant induction in plasma creatinine and Hcy levels, associated with tubular injury and neutrophil infiltration, and upregulation of pro-inflammatory cytokines and tubular apoptosis. Hcy treatment aggravated these renal damage and dysfunction by regulating cyclooxygenase-2 (COX-2), inhibitor of κB phosphorylation, and heme oxygenase-1. Consistently, Hcy treatment significantly increased expression of pro-inflammatory cytokines, glial fibrillary acidic protein, and COX-2 in the prefrontal cortex of IR mice. We conclude that Hcy treatment aggravated the renal dysfunction and enhanced IR-induced inflammatory cytokines and astrocyte activation in the brain. We propose that lowering plasma Hcy levels may attenuate neurological dysfunction found in patients with AKI.

## 1. Introduction

Acute kidney injury (AKI) is characterized by a sudden decline of glomerular filtration and tubular function, resulting in the accumulation of urea and other nitrogenous wastes and disruption of extracellular volume and electrolyte balance. AKI is determined by increased plasma creatinine, reduced urinary output and a duration less than 7 days [1]. AKI is found in 15~20% of patients admitted to the hospital and in over 50% of patients in intensive care units (ICU) [2]. Increased morality rates in patients with AKI are associated with several contributing factors, including vascular dysfunction, nephrotoxic exposures and systemic inflammation.

Gut-derived uremic toxins accumulate in the blood due to the impaired urinary excretion of metabolic wastes. Uremic toxins are identified in chronic kidney disease (CKD) patients, also found in human and experimental AKI [3]. Most frequently reported uremic toxins are indoxyl sulfate, phenyl sulfate, indole-3 acetic acid, uric acid, p-cresol, p-cresyl sulfate, and homocysteine, which have shown deleterious effects in various tissues [4]. These protein-bound uremic toxins are difficult to remove by dialysis due to their strong affinity to serum proteins [5]. Currently, research on identifying uremic toxins contributing to dysfunction of multiple organs in patients with AKI is actively conducted because a significant portion of ICU patients have complications of shock, sepsis, trauma, and surgery [6].

Uremic toxins are strong inducers of oxidative stress, exerting inflammatory mediators. In brain regions, this may be directly associated with neurovascular injury, astrocyte activation, and neuronal dysfunction, which contributes to cognitive disorders and poor stroke recovery in CKD patients [7]. AKI is associated with brain inflammation due to disruption of the blood–brain barrier, where infiltration of inflammatory mediators and uremic toxins occurs [8], which ultimately leads to brain dysfunction [9]. Elevated homocysteine (Hcy) is common in CKD and observed in 85% of dialysis patients [10]. Particularly, Hcy displays a deleterious effect in the brain by promoting neurovascular dysfunction, leukocyte recruitment [11] and glial activation [12], as well as exerting a direct neurotoxic role [13,14]; thus, Hcy is considered as a marker for neurodegenerative diseases [15]. However, direct evidence showing interventions to lower plasma Hcy levels to protect animals or patients with AKI from brain damage is still lacking.

In this study, we investigated the effect of Hcy on brain inflammatory responses induced by AKI in a mouse model of renal IR. Hcy treatment aggravated the renal dysfunction and enhanced IR-induced inflammatory cytokines and astrocyte activation in the regions of prefrontal cortex. We propose that lowering plasma Hcy levels in AKI patients may attenuate brain damage in patients complicated with neurological disorders.

## 2. Materials and Methods

### 2.1. Animals

Wild type (WT) C57BL/6 mice were purchased from Koatech Co. (Pyeongtaek, Republic of Korea) and were maintained in the animal facility at Gyeongsang National University. All animal experiments were approved by the Institutional Board of Animal Research at Gyeongsang National University and performed according to the National Institutes of Health guidelines for laboratory animal care (GNU-200612-M0036; 29 June 2020). Mice were housed with an alternating 12 h light/dark cycle and provided freely with water and standard chow.

### 2.2. Mouse Model of Renal IR Injury

Male mice (7-week old) were habituated for 1 week and randomly divided into 4 groups; (1) sham mice (*n* = 12), (2) mice subjected to renal IR (IR mice; *n* = 12), (3) mice treated with Hcy (*n* = 12), and (4) IR mice treated with Hcy (*n* = 12). Hcy (0.2 mg/kg; Sigma-Aldrich, St. Louis, MO, USA) was dissolved in saline and injected intravenously immediately after renal ischemia. The mice were anesthetized with zoletil (0.5 mg/kg; Virbac Laboratories, Carros, France), and placed supine on a heating pad under a heat lamp to maintain body temperature at 37 °C. The right and left renal pedicles were clamped with microaneurysm clips. After 25 min of ischemia, the clips were removed to allow reperfusion, and abdomen was closed by suture. Mice were sacrificed 24 h after reperfusion, and the blood, kidney, and brain tissues were collected. Blood was collected from an inferior vena cava using a heparinized syringe, and centrifuged at 3000× *g* for 20 min, and the supernatants were stored at −80 °C. The kidney, hippocampus and prefrontal cortex were rapidly frozen in liquid nitrogen and stored at −80 °C. The kidney was fixed in 10% buffered formalin and the brain was perfused transcardially (*n* = 4 each group) with heparinized saline followed by 4% paraformaldehyde.

### 2.3. Biochemical Assays

Plasma creatinine was measured using a direct colorimetric Jaffe method, and the levels were determined by a spectrophotometer (Shimadzu UV-1800 spectrophotometer, Tokyo, Japan). Plasma Hcy levels were measured by a mouse Hcy ELISA kit (MBS706003; MyoBioSource, San Diego, CA, USA) according to the manufacturer’s instructions.

### 2.4. Hematoxylin and Eosin (H&E) and Nissel Staining

The formalin-fixed kidney tissues were embedded in paraffin and 5 μm-thick sections were prepared and stained with H&E (Sigma-Aldrich) by a standard protocol. The renal injury scores were determined semi-quantitatively as previously described [16]. The PFA-fixed brains were sequentially immersed in 15% and 30% sucrose solutions at 4 °C until the tissues sank to the bottom of the container. The brains frozen in OCT compound (Sakura Finetek USA Inc., Torrance, CA, USA), were cut into 30-µm thick coronal sections. The brain sections were processed for Nissl staining (ab246816; Abcam, Cambridge, UK) by a standard protocol. All images were visualized with a CKX41 light microscope (Olympus, Tokyo, Japan).

### 2.5. Immunohistochemistry (IHC) Staining

The paraffin-fixed kidney sections were deparaffinized, rehydrated, and antigen-retrieved in sodium citrate buffer (10 mM, pH 6.0) for 20 min. The kidney sections were blocked in 10% normal horse serum and incubated with a primary antibody for Ly-6B.2 (MCA771GA; Bio-Rad, Hercules, CA, USA) overnight at 4 °C. The free-floating brain sections were incubated with primary antibody for GFAP (Z033429; Agilent Dako, Santa Clara, CA, USA) overnight at 4 °C. After washing of unbound antibodies, the sections were incubated with a biotinylated secondary antibody (Vector Laboratories, Burlingame, CA, USA) for 1 h at room temperature. Then, the sections were incubated in avidin-biotin-peroxidase complex solution (ABC solution; Vector Laboratories) for 30 min and developed by using a 3,3’-diaminobenzidine (DAB) Peroxidase Substrate Kit (Vector Laboratories, Burlingame, CA, USA). Finally, the sections were counterstained with hematoxylin and analyzed using a CKX41 light microscope (Olympus, Tokyo, Japan).

### 2.6. Terminal Deoxynucleotidyl Transferase dUTP Nick-End Labeling (TUNEL) Assay

TUNEL staining was performed using an In Situ Cell Death Fluorescein Detection Kit (Roche Molecular Biochemicals, Mannheim, Germany) according to the manufacturer’s instructions. The images were captured using Fluoview 1000 (IX-81) confocal microscope (Olympus, Tokyo, Japan).

### 2.7. Western Blot Analysis

The tissues were homogenized in radio-immunoprecipitation assay (RIPA) buffer with protease inhibitors (Thermo Fisher Scientific, Waltham, MA, USA), sonicated and incubated for 20 min on ice as described previously [17]. After centrifugation, the supernatant was transferred to a clean tube and the protein concentration was determined using a PierceTM BCA Protein Assay Kit (Thermo Fisher Scientific, Waltham, MA, USA). The protein lysates were separated using SDS-PAGE and transferred to PVDF membranes and blocked with 5% skim milk. The membranes were incubated with primary antibodies against COX-2 (cat# 12282), p-IκB (cat# 2589), IκB (cat# 9242), HO-1 (cat# 5061), caspase-3 (cat# 9662) from Cell Signaling Technology (Danvers, MA, USA), iNOS (sc-651; Santa Cruz Biotechnology, Dallas, TX, USA), and β-actin (A5441; Sigma-Aldrich, MO, USA) in the blocking solution at 4 °C overnight. Next, the membranes were incubated with the appropriate horseradish peroxidase (HRP)-conjugated secondary antibodies (Bio-Rad, Hercules, CA, USA) at room temperature for 1 h and then visualized with the ECL substrate (Bio-Rad, Hercules, CA, USA). The ChemiDoc XRS + System (Bio-Rad, Hercules, CA, USA) was used to evaluate the density of protein bands, and relative protein levels were quantified using Image LabTM software (Bio-Rad, Hercules, CA, USA) according to the manufacturer’s instructions.

### 2.8. Reverse Transcription and Quantitative PCR Analysis

The total RNA was extracted with Trizol (Invitrogen, Carlsbad, CA, USA) and converted into cDNA using the RevertAid Reverse Transcription System (Thermo Fisher Scientific, Waltham, MA, USA) according to the manufacturer’s protocol. Quantitative PCR was performed on the CFX Connect real-time PCR System using iQ SYBR Green Supermix (Bio-Rad, Hercules, CA, USA). Relative mRNA levels were normalized to those of GAPDH for each gene. The primer sequences are listed in Table 1.

### 2.9. Statistical Analysis

Statistical significance was determined using one-way ANOVA analysis, followed by Bonferroni’s multiple comparisons (GraphPad Prism 7 Software, v.7.00, La Jolla, CA, USA). Data were expressed as the mean ± SEM. * *p* < 0.05 vs. sham mice, and # *p* < 0.05 vs. IR mice.

## 3. Results

### 3.1. Homocysteine Exacerbates Renal IR-Induced Renal Damage

**Figure 1:**
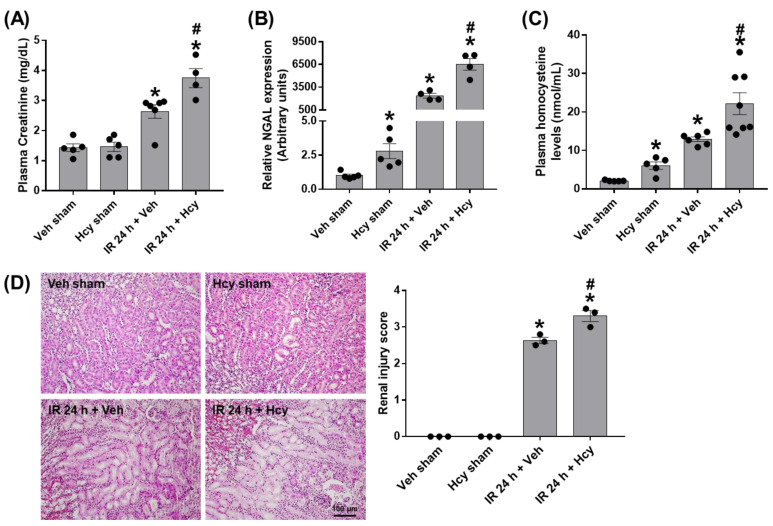
Homocysteine (Hcy) exacerbates renal IR-induced renal damage. C57BL/6 mice were subjected to renal ischemia for 25 min, immediately intravenously injected with saline or Hcy (0.2 mg/kg), and sacrificed 24 h after reperfusion. Blood, kidney and brain samples were collected from sham and IR mice treated with saline or Hcy (*n* = 12). Plasma creatinine and Hcy levels and renal expression of neutrophil gelatinase-associated lipocalin (NGAL) were determined (**A**–**C**). Kidney sections were stained with H&E and tubular damages were scored as described (**D**). Data are presented as the mean ± SEM. * *p* < 0.05 vs. sham mice; # *p* < 0.05 vs. IR mice. Black dots indicate individual data points. Scale bar, 100 μm.

To investigate the effect of homocysteine (Hcy) on renal function in IR mice, plasma creatinine levels were measured in sham and mice subjected to renal IR treated with saline or Hcy. At 24 h after reperfusion, the levels were significantly increased, and elevated further by Hcy treatment (Figure 1A). Consistently, the mRNA expression of NGAL, a kidney injury marker, was significantly increased by Hcy treatment in sham and IR mice (Figure 1B). The exacerbating effect is related with plasma Hcy levels (Figure 1C), and we found a positive correlation (r = 0.8935, *p* < 0.0001) between NGAL and plasma Hcy. The tubular injury induced by renal IR also aggravated by Hcy treatment as shown in Figure 1D. Renal injury was determined by scoring the percentage of tubules displaying tubular necrosis, sloughing of tubular epithelial cells or loss of brush borders, cast formation and tubular dilatation as previously described [16].

### 3.2. Hcy Exacerbates Renal IR-Induced Inflammation and Tubular Apoptosis in the Kidney

**Figure 2:**
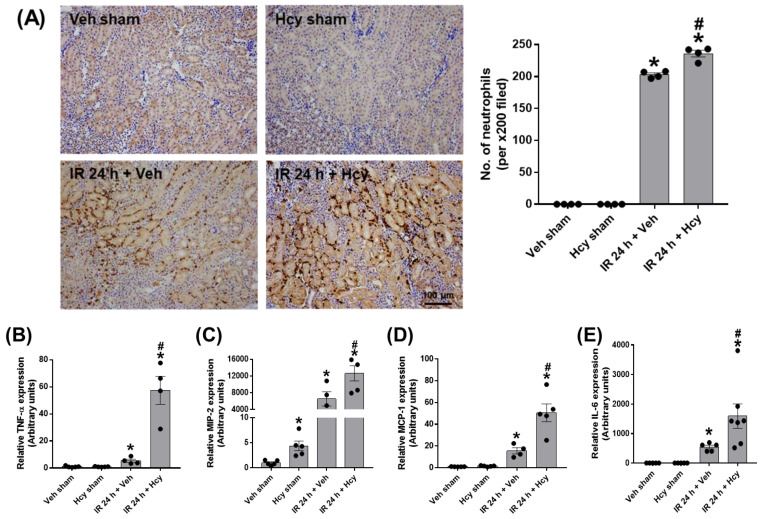
Hcy exacerbates renal IR-induced neutrophil infiltration and pro-inflammatory cytokine elevation in the kidney. Neutrophil infiltration was assessed by polymorphonuclear leukocyte immunohistochemical staining using Ly-6B.2 antibody and the numbers of stained neutrophils per 200× field image were counted (**A**). The mRNA expression of pro-inflammatory cytokines (TNF-α, MIP-2, MCP-1, and IL-6) was determined in the kidney by real-time PCR analysis (**B**–**E**). Data are presented as the mean ± SEM. * *p* < 0.05 vs. sham mice; # *p* < 0.05 vs. IR mice. Black dots indicate individual data points. Scale bar, 100 μm.

**Figure 3:**
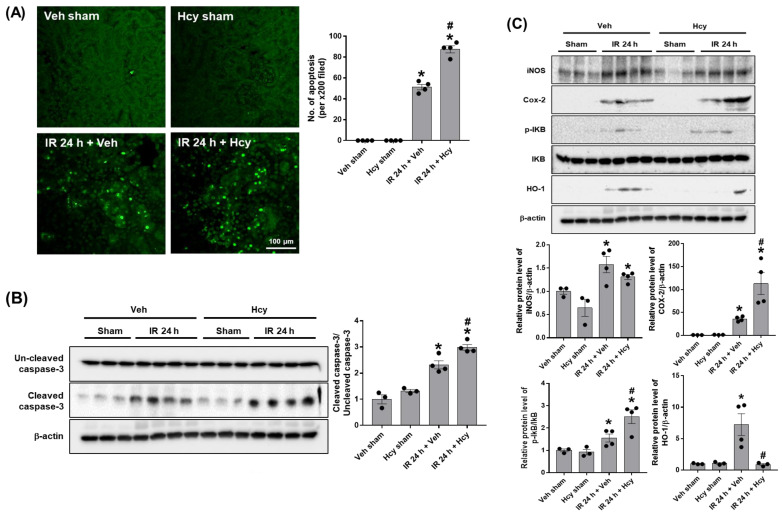
Hcy exacerbates renal IR-induced tubular apoptosis in the kidney. The renal tubular apoptosis was determined by TUNEL staining and the numbers of apoptotic cells per 200× field image were counted (**A**). The expression of uncleaved and cleaved caspase-3 was determined by Western blot analysis and the quantification was shown (**B**). The expression levels of iNOS, Cox2, p-IκB, IκB, and HO-1 were determined by Western blot analysis and the quantirication was shown (**C**). Data are presented as the mean ± SEM. * *p* < 0.05 vs. sham mice; # *p* < 0.05 vs. IR mice. Black dots indicate individual data points. Scale bar, 100 μm.

To assess the effect of Hcy on tubular inflammation, we performed polymorphonuclear leukocyte (PMN) staining. IR mice showed a significant neutrophil infiltration, which was further elevated by Hcy treatment (Figure 2A). Expression levels of pro-inflammatory cytokines, TNF-α, MIP-2, MCP-1, and IL-6 were significantly increased in IR mice and Hcy treatment exacerbated the cytokine induction (Figure 2B–E). To assess the effect of Hcy on tubular apoptosis, we performed TUNEL staining. IR mice showed a significant induction of tubular apoptosis which was highly elevated by Hcy treatment (Figure 3A). Expression of cleaved caspase-3 was increased in IR mice, which was further elevated by Hcy treatment as shown in Western blot analysis (Figure 3B). The levels of cyclooxygenase-2 (COX-2) and inhibitor of κB (IκB) phosphorylation were increased, but inducible nitric oxide synthase (iNOS) levels were not changed by Hcy treatment (Figure 3C). Previously, hyperhomocysteinemia accelerates AKI to CKD progression by downregulating heme oxygenase-1 (HO-1) expression [18]; consistently, the HO-1 levels were reduced by Hcy treatment in IR mice (Figure 3C). The results indicate that Hcy exacerbate the renal inflammation and tubular apoptosis through COX-2, IκB, and HO-1 signalling in IR injury.

### 3.3. Hcy Induces Astrocyte Activation and Inflammation of the Prefrontal Cortex in Renal IR

**Figure 4:**
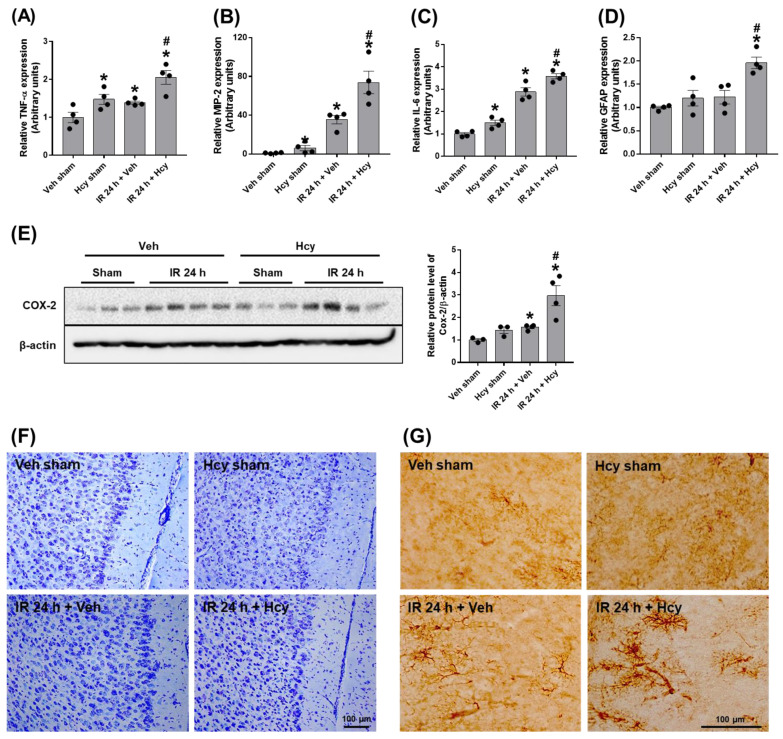
Hcy exacerbates renal IR-induced astrocyte activation and pro-inflammatory cytokine elevation in the prefrontal cortex. The mRNA expression of pro-inflammatory cytokines (TNF-α, MIP-2, and IL-6) and glial fibrillary acidic protein (GFAP) was determined by real-time PCR analysis (**A**–**D**). The COX-2 expression levels were determined by Western blot analysis and the quantification was shown (**E**). The brain sections were processed for Nissel staining and the representative images of precrontal cortex were shown (**F**). Astrocyte activation was assessed by immunohistochemical staining using GFAP antibody (**G**). Data are presented as the mean ± SEM. * *p* < 0.05 vs. sham mice; # *p* < 0.05 vs. IR mice. Black dots indicate individual data points. Scale bar, 100 μm.

A previous study has shown that cognitive impairment found in CKD is associated with uremic toxin-induced blood-brain-barrier (BBB) disruption [19], which suggests that circulating peripheral inflammatory cytokines and uremic toxins easily penetrate the BBB and worsen the brain inflammation. To assess the effect of Hcy on the brain inflammation following renal IR, we measured the mRNA levels of pro-inflammatory cytokines, TNF-α, MIP-2 and IL-6 in the prefrontal cortex (PFC). The levels were significantly increased in IR mice, and elevated further by Hcy treatment (A expression of glial fibrillary acidic protein (GFAP), a marker of astrocyte activation, was significantly increased in Hcy-treated IR mice although the expression was not induced in IR mice (Figure 4D). Expression of COX-2 was also upregulated by Hcy treatment in IR mice (Figure 4E). However, neuronal loss or histological changes were not observed in IR or Hcy-treated IR mice as shown in Nissel staining (Figure 4F). Consistent with the mRNA induction of GFAP, immunochemical staining of GFAP showed significant levels of astrocyte activation by Hcy treatment in IR mice (Figure 4G). In add ition, we performed experiments to show the effect of Hcy on hippocampal inflammation. We found no significant induction of mRNA expression in TNF-α, MIP-2 and IL-6 and no changes in GFAP levels at 24 h post reperfusion (Supplementary Figure S1A). However, there was a significant increase in mRNA expression in TNF-α, MIP-2 and IL-6 at 48 h post reperfusion (Supplementary Figure S1B). IR mice did not show hippocampal death or discernible astrocyte activation by Hcy treatment (Supplementary Figure S2A,B). In addition, there was no changes in the mRNA expression of neuronal injury markers, S100-β, neuron specific enolase (NSE), and myelin basic protein (MBP) in the hippocampus (Supplementary Figure S2C). These indicate that the cytokine increase and glial activation occurs prior to neuronal death in the brain of IR mice.

## 4. Discussion

We investigated the effect of uremic toxin, Hcy on brain inflammation in mice subjected to renal IR. First, we found that plasma creatinine and NGAL expression levels were correlated with enhanced plasma Hcy levels in IR mice. Hcy treatment aggravated tubular damage, renal inflammation and apoptosis. The observed renal injury was associated with altered expression of COX-2, IκB phosphorylation, and HO-1. Second, we found that in IR mice, the PFC exhibited a significant increase of pro-inflammatory cytokines and GFAP expression, indicative of astrocyte activation, by Hcy treatment. We propose that lowering plasma Hcy levels in AKI patients may have therapeutic benefit in the development of neurological disorders.

Hcy is an intermediate of methionine metabolism and elevated in patients with renal diseases, due to the impaired glomerular filtration and tubular function. Elevated plasma Hcy contributes to the progression of end-stage renal disease (ESRD). In human, the normal levels of Hcy are 5–15 uM, and elevated Hcy levels considered as significant risk factor are 30–50 uM, which is 3–5 times higher than normal levels, prevalent in 85–100% of renal patients [20]. Hyperhomocysteine (HHcy) predicts renal functional decline in hypertensive adults [21], and accelerates AKI to CKD progression [22]. The pathogenic mechanisms of Hcy include oxidative stress, inflammation, endoplasmic reticulum stress [20], and mitochondrial damage [23].

In rats subjected to ischemia, the impaired Hcy metabolism results in Hcy accumulation in the kidney and subsequently in the plasma. Renal administration of anti-Hcy antibodies reduced IR-induced oxidative stress and apoptosis after 1 h of reperfusion, but not after 24 h of reperfusion [24], which suggests a detrimental effect of Hcy directly in the ischemic kidney, followed by in remote organs in reperfusion period. In this study, brain injury was assessed by expression of pro-inflammatory cytokines and GFAP after 24 h of reperfusion in IR mice where Hcy were treated immediately after ischemia. Astrocyte activation in the PFC was prominent in IR mice treated with Hcy but not with vehicle. Hcy is metabolized immediately in normal healthy conditions, but is accumulated in renal diseases where multiple organ dysfunction is not restored by antagonizing or removing Hcy afterwards. Thus, the therapeutic interventions to lower Hcy should be made early in AKI patients before plasma Hcy accumulates in high levels.

Previously, Hcy was shown to increase the expression and secretion of chemokines, MCP-1 and IL-8 from human monocytes through upregulation of intracellular ROS originated from NADH oxidase [11]. The deleterious effects of Hcy were mediated by BBB disruption and vascular inflammation, shown by an increased expression of intracellular adhesion molecule 1 (ICAM-1), matrix metalloproteinase (MMP)-2 and -9, and GFAP in the brain [25]. Interestingly, Hcy also exerts a direct neurotoxic role by overstimulating N-methyl-D-aspartate receptors [13,14]. In fact, epidemiological studies have confirmed that elevation in plasma Hcy precedes the development of dementia and has an inverse relation with cognitive performance in older persons, considering Hcy as a marker for neurodegeneration [15].

Astrocyte activation is not shown in hippocampus in IR mice treated with Hcy in this study; however, we found a significant induction in mRNA expression of TNF-α, MIP-2 and IL-6 at 48 h post reperfusion. In this study, we found no neuronal death, implying that cytokine increases and glial activation may occur prior to neuronal injury. Previous studies also suggest that increases in TNFα and IL-1β have been observed prior to neuronal death, and the glial activation of immune factors such as TNF causes neuronal death [26,27]. Consistently, in a mouse model of severe AKI by inducing renal ischemia for 60 min, marked hippocampal changes were reported; pyknotic neurons, glial activation, cytokine/chemokine induction, and impaired BBB integrity [8]. Locomotor activity was also reduced in severe AKI [8] and a higher incidence of cognitive impairment and dementia was found in AKI patients [28]. Uremic encephalopathy is defined as cerebral dysfunction due to the accumulation of uremic toxins resulting from acute or chronic renal failure, increasing morbidity and mortality of renal patients. Thus, other uremic toxins found in the brain can be targeted therapeutically in future pathogenic study to prevent cognitive decline [6,7].

## 5. Conclusions

In conclusion, we found that Hcy treatment aggravated the renal dysfunction and enhanced IR-induced inflammatory cytokines and astrocyte activation in the brain. We propose that lowering plasma Hcy levels may attenuate the brain inflammation found in AKI patients with AKI, and may alleviate a risk of neurodegeneration. We suggest an anti-Hcy treatment such as injecting anti-Hcy antibodies into IR mice as a therapeutic potential.

## Figures and Tables

**Table 1:** The primer sequences used in this study.

Genes	Forward Primers (5′-3′)	Reverse Primers (5′-3′)
GAPDH	GTGGCAAAGTGGAGATTGTTG	TTGACTGTGCCGTTGAATTTG
GFAP	AGAAAGGTTGAATCGCTGGA	CGGCGATAGTCGTTAGCTTC
IL-6	CCAATTCATCTTGAAATCAC	GGAATGTCCACAAACTGATA
MCP-1	ACCTTTGAATGTGAAGTTGA	CTACAGAAGTGCTTGAGGTG
MIP-2	AGAGGGTGAGTTGGGAACTA	GCCATCCGACTGCATCTATT
NGAL	CACCACGGACTACAACCAGTTCGC	TCAGTTGTCAATGCATTGGTCGGTG
TNFα	CATATACCTGGGAGGAGTCT	GAGCAATGACTCCAAAGTAG

## Data Availability

Not available.

## Supplementary Materials

The following supporting information can be downloaded at: https://www.mdpi.com/article/10.3390/biomedicines10123048/s1, Figure S1: The effect of Hcy on renal IR-induced inflammation at 24 h and 48 h post-reperfusion in the hippocampus, Figure S2: Nissel staining, astrocyte activation and the mRNA expression of neuronal injury markers in the hippocampus.
