# Supplementary material for: The Uremic Toxin Homocysteine Exacerbates the Brain Inflammation Induced by Renal Ischemia-Reperfusion in Mice
Source: Biomedicines. 2022 Nov 25;10(12):3048. doi: 10.3390/biomedicines10123048 (PMC9775228; doi:10.3390/biomedicines10123048)
Supplement: Supplementary file 1 [file biomedicines-10-03048-s001.zip › biomedicines-1998340-supplementary.pdf]

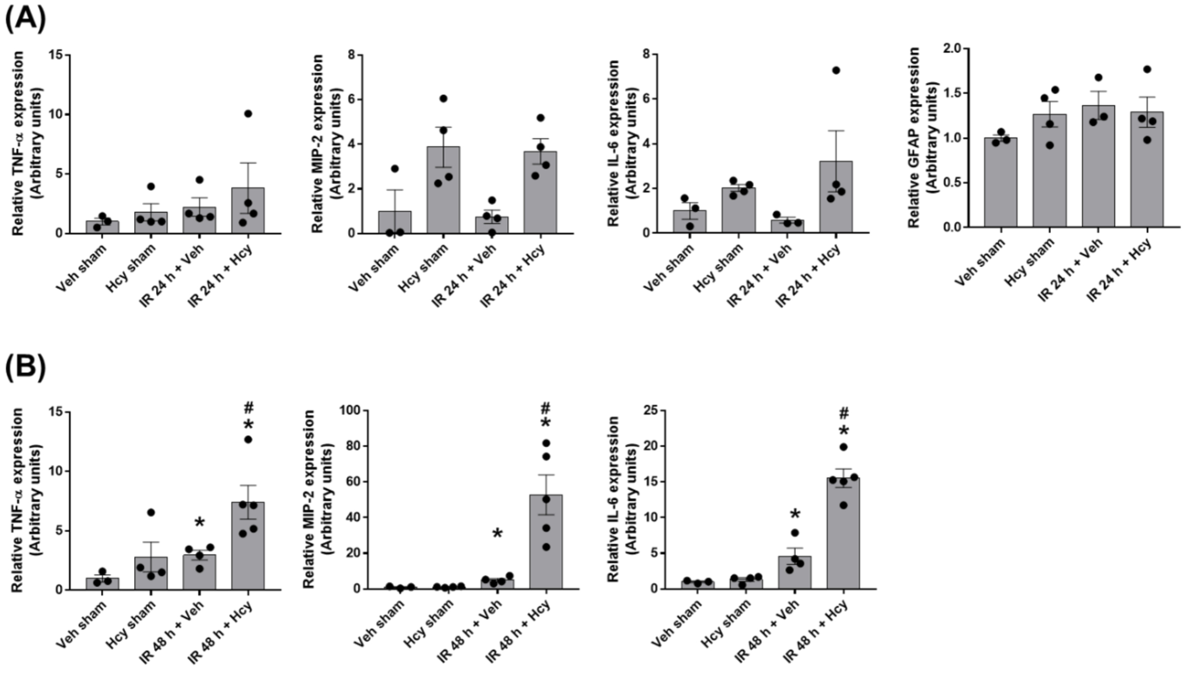

Figure S1. The effect of Hcy on renal IR-induced inflammation at 24h (A) and 48h (B) post-reperfusion in the hippocampus. The mRNA expression of pro-inflammatory cytokines (TNF-α, MIP-2, and IL-6) and glial fibrillary acidic protein (GFAP) was determined by real-time PCR analysis. Data are presented as the mean ± SEM. *p <0.05 vs sham mice; #p <0.05 vs IR mice. Black dots indicate individual data points

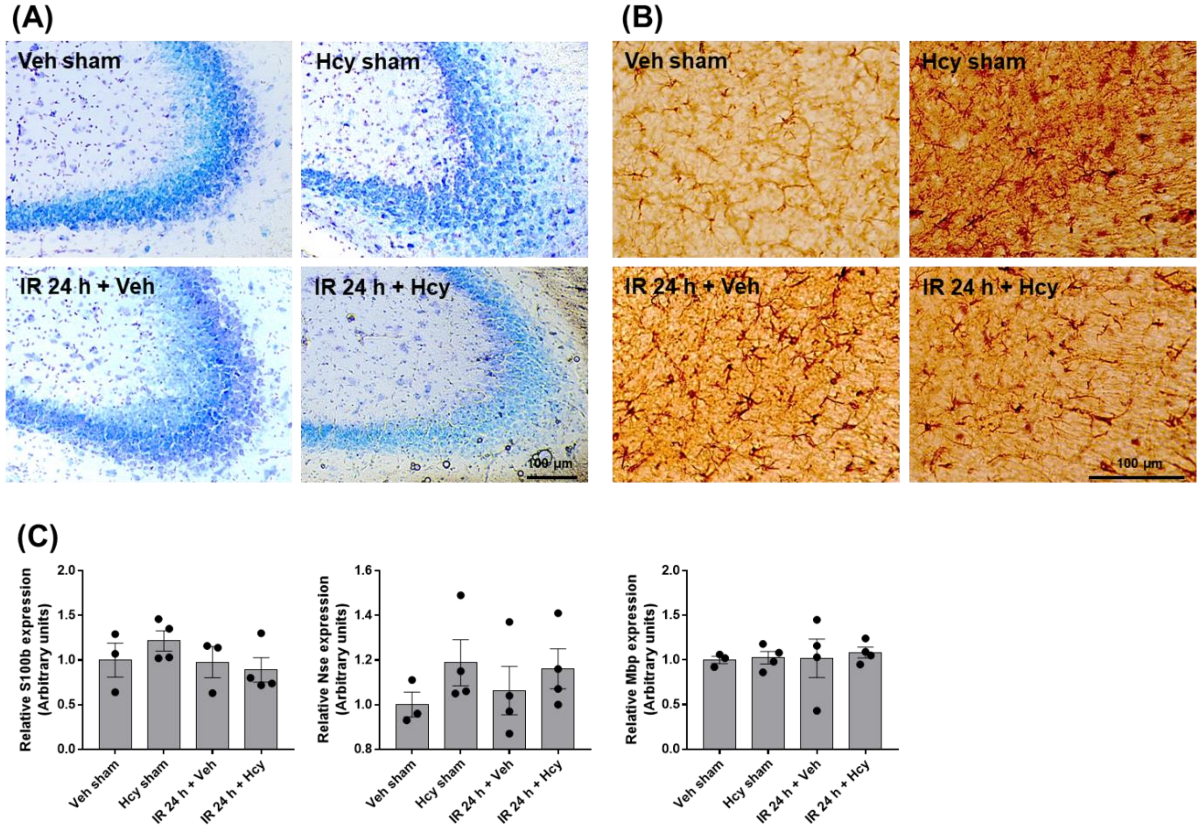

Figure S2. The brain sections were processed for Nissel staining and the representative images of hippocampus were shown (A). Astrocyte activation was assessed by immunohistochemical staining using GFAP antibody (B). The mRNA expression of neuronal injury markers, S100-β, neuron specific enolase (NSE), and myelin basic protein (MBP) in the hippocampus was determined by real-time PCR analysis (C). Scale bar, 100 µm. Black dots indicate individual data points.
